# What causes type 1 diabetes? Lessons from animal models

**DOI:** 10.1111/j.1600-0463.2011.02765.x

**Published:** 2011-07

**Authors:** Karsten Buschard

**Affiliations:** From Bartholin Instituttet, Rigshospitalet, Copenhagen and Department of Veterinary Disease Biology, Faculty of Life Sciences, University of CopenhagenFrederiksberg, Denmark

## Abstract

To study type 1 diabetes (T1D), excellent animal models exist, both spontaneously diabetic and virus-induced. Based on knowledge from these, this review focuses on the environmental factors leading to T1D, concentrated into four areas which are: (1) The thymus-dependent immune system: T1D is a T cell driven disease and the beta cells are destroyed in an inflammatory insulitis process. Autoimmunity is breakdown of self-tolerance and the balance between regulator T cells and aggressive effector T cells is disturbed. Inhibition of the T cells (by e.g. anti-CD3 antibody or cyclosporine) will stop the T1D process, even if initiated by virus. Theoretically, the risk from immunotherapy elicits a higher frequency of malignancy. (2) The activity of the beta cells: Resting beta cells display less antigenicity and are less sensitive to immune destruction. Beta-cell rest can be induced by giving insulin externally in metabolic doses or by administering potassium-channel openers. Both procedures prevent T1D in animal models, whereas no good human data exist due to the risk of hypoglycemia. (3) NKT cells: According to the hygiene hypothesis, stimulation of NKT cells by non-pathogen microbes gives rise to less T cell reaction and less autoimmunity. Glycolipids presented by CD1 molecules are central in this stimulation. (4) Importance of the intestine and gliadin intake: Gluten-free diet dramatically inhibits T1D in animal models, and epidemiological data are supportive of such an effect in humans. The mechanisms include less subclinical intestinal inflammation and permeability, and changed composition of bacterial flora, which can also be obtained by intake of probiotics. Gluten-free diet is difficult to implement, and short-term intake has no effect. Regarding the onset of the T1D disease process, slow-acting enterovirus and gliadin deposits are speculated to be etiological in genetically susceptible individuals, followed by the mentioned four pathogenetic factors acting in concert. Neutralization of any one of these factors is capable of stopping T1D development, as lessons are learned from the animal models.

## Introduction

In spite of intensive research during the last decades, the question in the title cannot be answered briefly, precisely, or without any doubt. However, several pieces of evidence have been obtained and the solutions might not be far away. Some aspects have been highlighted to a greater extent than others and, therefore, it seems necessary to review the data and indications available in a new approach.

Type 1 diabetes (T1D) is a disease for which good animal models exist. These include the spontaneously diabetic BB rats and NOD mice, as well as virus-induced diabetes in mice; see (1). However beneficial the models may be, it is essential that the information obtained is evaluated critically and is related to human data.

T1D is to some extent genetically influenced, and mostly by certain MHC types. However, 90% of T1D cases have no first-degree relatives, and the pairwise concordance rate for monozygotic twins is described to be 27% (2). Whether epigenetic studies in the coming years might expand the genetic component is uncertain at present. In any event, T1D is a disease in which the environment plays a major role.

It is in good accordance with the partially uninherited nature of T1D that the incidence of the disease during the last 3–4 decades has increased substantially, mostly in highly developed countries with a western lifestyle. In these societies, and especially in Finland, T1D is seen in up to 2% of all individuals during their lifetime. This is an unusually high incidence for a potentially deadly disease, only comparable with that of rheumatoid arthritis. Autoimmunity is breakdown of tolerance, and interestingly the organ systems both of insulin production (beta cells) and of physical body movement (joints) are less developed at birth and thereby less known by the immune cells, due to the special human problem of creating the big brain. Not until several weeks of age do the beta cells become glucose-sensitive, and not before one year are we able to walk.

This review will focus on four issues, which are all decisive for the development of the disease. For each of them it holds true that, in the animal models, T1D will not occur if the specific factor is neutralized. At the end of this paper, a list of the events related to the disease is given, and how these factors interact during the various phases of the T1D disease process is described.

## I. The thymus-dependent immune system

The modern era in T1D research began in 1965 when Gepts (re)discovered the insulitis process in pancreatic tissues from T1D patients (3). Although T cells were found to be present *in situ* in the islets, T1D was not accepted as an autoimmune disease by all researchers. Indeed, T1D was seen as being the result of a T cell defect, which failed to destroy a destructive virus. Not until the discovery of islet cell antibodies (ICA) (4), was T1D categorized as being autoimmune, but the mechanisms were still widely unknown.

Studies using passive transfer focused on the thymus-dependent immune system (5). Even virus-induced T1D, using EMC-M virus, was not seen in nude mice but only in thymus-competent normal mice (6–8). Also, if the T cells were inhibited by cyclophosphamide, EMC virus did not induce diabetes (9) and, more specifically, the effect seems to be dependent on helper T cells (10). Later studies along the same line showed a beneficial effect on T1D by immune suppression e.g. using cyclosporine (11, 12) or CD3 antibodies (13). This kind of treatment would not have been possible to institute if the etiology had involved a toxic virus. Recent virus studies show persistent presence of virus in the beta cells (14). Thus, T1D is a T cell-dependent disease, and if T cells are inactivated, T1D will not develop.

During the natural history of T1D, T cell activity develops against more and more beta-cell epitopes, which is often referred to as antigen spreading. The antigens include insulin, glutamic acid decarboxylase (GAD), IA2, zinc-transporter protein, and others, most likely also some unknowns. The presence of both effector T cell reactions and autoantibodies can be detected. The antibodies are highly valued as prognostic markers. In children, the first to arrive are typically insulin autoantibodies (IAA), but in adolescent and adult T1D patients, GAD Abs are even more frequent. IA2 and zinc-transporter Abs are well correlated with the progress of the disease. Being positive for only one autoantibody is quite safe, but having two or more Abs, especially in high titers, is decisive for a T1D development. In an important case study, T1D was seen in a patient with severe B lymphocyte deficiency (15). This stresses that autoantibodies, no matter how well prognostic they may be, are of no pathogenic significance. Although, recently B lymphocytes have been demonstrated to be of some importance for islet graft rejection, probably as antigen presenting cells (16).

In contrast, effector T cells are for sure pathogenetic. T cell reactions against the mentioned beta-cell epitopes can be detected *in vitro*, and the results indicate a prognostic prediction; especially regarding the outcome of islet or pancreas transplantation, such a prediction is significant (17). T cell activity is influenced by regulator T (Treg) cells, which were earlier called suppressor T cells. Already in 1980, it was shown that suppression is impaired in patients with newly diagnosed T1D (18). This has been confirmed later, and it has been suggested that the defective regulation in T1D might be due to resistance of the effector T cells to respond to the Tregs (19). Thus, normal development of Treg cells occurs in response to islet antigens, but the reaction from the effector T cells is defective (20). The number of peripheral Treg cells seems not to be changed. Interestingly, regulator T cells are raised specifically but act unspecifically *in situ* at their relevant placement. This is probably the reason why regulator T cells directed against different antigens are all active in diminishing the destructive insulitis process, and why vaccination studies using different antigens have given comparable results in delaying C-peptide deterioration.

The mechanisms by which T cells destroy the beta cells have been studied extensively. Bendtzen suggested in 1986 that IL-1β probably together with IFNγ and TNFα induced necrosis (21). Later also apoptosis was nominated to be induced by this mechanism. Support for this theory is coming additionally from studies in BB rats. On the other hand, in NOD mice it appears to be quite clear that the beta cells are hurt by perforin and granzymes (22, 23).

Hormone producing cells might be especially sensitive to autoimmune diseases due to the fact that these cells open themselves up during the secretion of their specific molecules. This holds true for adrenal cortex, thyroid glands, and beta cells. That diabetes is the most common disease – at least in younger age groups – might be associated with the protein nature of insulin, whereas the other glands produce smaller molecules that may be less antigenic. Also, the pituitary gland produces protein hormones but it is protected behind the blood brain barrier, which might be the reason for much less autoimmunity associated with this tissue.

It might well be imagined that not every single insulin molecule out of several billions produced is totally correct, and therefore could elicit an antigenic reaction. It would therefore seem appropriate that there are anti-inflammatory mechanisms associated with the beta cells. We have found that sulfated beta-galactosyl ceramide (sulfatide), which is associated with insulin and which is present at the surface of the beta cells, acts against inflammation. Sulfatide decreases cytokine (24) and chemokine (25) secretion, it reduces the destructive actions of cytokines on beta cells, and it stimulates regulatory NKT cells (26). Furthermore, sulfatide inhibits diabetes development in NOD mice (27), and presence of sulfatide *in vitro* resulted in greatly reduced proliferation of an insulin-specific T-cell clone (28). Sulfatide is a glycosphingolipid produced in the beta cells, and its association with diabetes has been reviewed in 2005 (29).

Vitamin D can modify the immune response, and the relative lack of this vitamin in northern countries fits well with the north-south gradient of T1D incidence. Furthermore, vitamin D has been shown to have a protective effect on IL-1 damage of beta cells (30). Also, inhibition of insulitis and diabetes in NOD mice has been demonstrated (31, 32). On the other hand, in humans the level of vitamin D in plasma is not associated with development of beta-cell autoimmunity (Norris JM, personal communication).

### Suggestions for treatment

Trials are running in order to suppress the T cell-dependent immune system, either unspecifically or by suppressing specific reactions against certain beta-cell epitopes. The former includes treatment with anti-CD3 Ab (33) and among the latter are antigen tolerization against GAD (34) or insulin, either given orally (35) or as a proinsulin vector injected intramuscularly (36). The mechanism of action seems to be expansion of regulator T cells, which then act upon the insulitis process (37). The effects of the various trials are comparable; none of them stop beta-cell destruction as measured by C-peptide concentration, but the disease process is delayed by one to three years. Among the new compounds, sulfatide might be considered for trials in as much as it has other desirable effects that might help in prevention of T1D (29), see below.

The theoretical *risk* of the unspecific depression of the immune system, as small as it might be, is an increased incidence of cancer. This is well known in organ transplantation (e.g. heart or kidney) using relatively high dosage of immunotherapy. Whether it actually plays a role in T1D immunomodulation is too early to judge. For the specific epitope vaccination, the risk is always that aggressive T cells are stimulated to a higher degree than regulator T cells, with the consequence that the disease process is accelerated instead of being delayed.

## II. The activity of the beta cells

The importance of the activity of the beta cells has been suggested in 1985 (38) and reviewed in 1991 (39). Growing evidence suggests that the functional state of the beta cell plays a role in the pathogenesis of T1D. Increased incidence of diabetes has been described after increased insulin production and vice versa, and actual hyperinsulinemia has been observed in relation to the diabetogenesis. First-degree relatives with increased risk of T1D have been shown to display higher blood insulin concentrations (40). Furthermore, in the period before clinical diabetes is diagnosed, patients might have eaten food with a high glycemic index (41). In the last trimester of pregnancy, the beta cells are stressed by a highly increased insulin demand, partly due to an increased amount of counteracting hormones. During this trimester, development of true T1D is 3.8 times more frequent than in non-pregnant women (42). In the progression from being autoantibody-positive to having overt T1D, insulin resistance seems to be a risk factor (43, 44). This fits well with the finding that in BB rat litters, it is the heaviest rat that develops T1D first (45). This might have inspired to the accelerator hypothesis formulated by Wilkin (46), which says that increased weight gain in youngsters might accelerate a T1D development. The question can be raised as to what is most important: a high degree of stress or lack of rest? In a study using BB rats, food intake only every other or third day resulted in nearly the same weight gain but in less diabetes development. The beta-cell stress on the eating day seems to be of less significance than the beta-cell rest on the other days (47). In humans, metabolic improvements including increased plasma adiponectin have correspondingly been shown (48).

The size of the beta cell mass and the number of islets are known to vary considerably in rodents (49), which is probably also the case in humans, as reflected by the noticeable variation in normal values of C-peptide concentrations. This may be genetically determined, but influence from other factors cannot be ruled out, such as length of pregnancy for which shorter length predisposes slightly to T1D (50, 51), and birth by caesarean section which increases the T1D risk by 23% (52). It is an attractive idea, but unknown whether low islet mass, which might be more stressful for the individual beta cell, predisposes to T1D.

Pharmacologically, efforts have been made to induce beta-cell rest both in animal models and in human (pre)T1D. As is the case for other endocrine cells, the hormone production is decreased when the hormone in question is administered. Injections of insulin accordingly induce some degree of beta-cell rest, and the treatment regime in order to do so is termed *prophylactic insulin treatment* (53). This substantially reduces the incidence of T1D in BB rats (53). The finding has been repeated by several groups, and in an open pilot study human preT1D patients also benefitted from prophylactic insulin treatment by showing delayed disease progression (54). However, this could not be confirmed in the large, prospective Diabetes Prevention Program Trial (DPPT) where the treatment had no effect (55). Because of the fear of hypoglycemic events, the dose of insulin used was as low as 0.1 U/kg body weight (55). By comparison, the original study in BB rats used 15 U insulin/kg body weight (53). Actually, the very low human dose was later found not to work, either in BB rats or in NOD mice (56). The human dose used might give an immunological effect, which then was not enough for T1D prevention. Unfortunately, no pilot trial of dose-response was performed before the DPPT was started, and the dose was chosen according to the wish to avoid insulin shocks (which were not seen) and according to the availability of sponsored insulin.

Another way of inducing beta-cell rest is to activate the beta-cell potassium channels. This will then close the calcium channels, and no insulin secretion will occur. Indeed, such a treatment with diazoxide reduced development of T1D in BB rats (57). Also, in human T1D patients diazoxide treatment showed an effect in form of higher insulin secretion after one year compared to placebo-treated patients (58). Diazoxide given to children with T1D prolonged their remission period (59). Since diazoxide has undesirable side effects, the pharmaceutical industry has attempted to develop other drugs in order to obtain the same activation of potassium channels. Such a drug known as NN 414 showed a good effect against T1D in BB rats (60), but unfortunately the compound was later withdrawn due to its side effects on liver enzymes. Interestingly, nature itself has a potassium channel activator secreted by the beta cells together with insulin (61). This is sulfatide, which opens the K^+^ channels, and by this induces beta-cell rest for the individual beta cell (62, 63). Then, the next beta cell can take over and the first one can rebuild insulin granules close to the cell membrane, which are necessary for the immediate first-phase insulin response. Whether sulfatide due to this property is a candidate as a pharmacological compound is unresolved at the moment.

As mechanisms for the outcome of beta-cell rest, at least three possibilities have been suggested.

*First*, increased antigen expression (including both gangliosides and proteins) in beta cells with high activity (64) could facilitate destruction caused by the immune system. Thus, lower levels of specific antigens are expressed at the surface of a passive cell, which have been demonstrated for several antigens (65–67). Given the T cell dependent nature of T1D, this is probably of great importance. Autoimmunity is breakdown of self-tolerance, which should be established during fetal and neonatal life. An adult phenotype of beta cells is not achieved before weeks after birth, unless the baby is born to a diabetic mother or – in animal models – unless the beta cells have been stimulated to secrete insulin by e.g. arginine (68, 69). In both cases the beta cells are phenotypic adult instead of being the usual fetal type, and the risk of later development of T1D is reduced (68–70). This emphasizes the need for a good antigenic self response in a given tissue at the neonatal stage in order to avoid later breakdown of tolerance. Furthermore, a different immune response against adult compared to fetal beta cells has been shown (71). The principle of neonatal stimulation in order to diminish later autoimmunity has been extended successfully to the thyroid gland (72). It should be mentioned that to explain the lower risk of T1D in children born of diabetic mothers, compared to offspring of diabetic fathers (70), a beneficial effect of transmission of maternal islet antibodies has also been suggested (73).

*Second*, increased susceptibility to the toxicity of cytokines (74) or mononuclear spleen cells from diabetic BB rats or NOD mice (75) has been shown for active as compared to passive beta cells. In line with this is the finding that the NN 414 potassium activator protects against cytokine-induced apoptosis of beta cells *in vitro* (76).

*Third*, several genes have been described to change expression as a function of beta-cell activity (77–79); this might well influence beta-cell resistance.

### Suggestions for treatment

One way to induce rest of a hormone-producing cell is to provide the body with the same hormone externally. For beta cells, this can be performed by *prophylactic insulin treatment* using metabolic doses. Hopefully, a serious trial with a serious insulin dosage will not be too far away.

Beta-cell rest can also be induced by *treatment with potassium channel activators*. As mentioned, this has been tried with diazoxide and the commercial derivative NN 414. Although there were perfect results in animal studies, and even some success in humans, treatment with these compounds has been stopped due to pharmacological side effects. Treatment with sulfatide might be a new possibility.

A third method for induction of beta-cell rest could be *diet with low glycemic index* combined with exercise. This kind of treatment, known partly from the days before the insulin era, is not realistic to be implemented on a large scale.

The *risk* with treatment for beta-cell rest might be some degree of atrophy of the islet-cell volume and induction of hypoglycemia. The fear of the latter might be eliminated by good education of the patients.

## III. The innate immune system

In humans and other higher organisms, bacteria are, if necessary, attacked by granulocytes, macrophages, and NK(T) cells, which define the innate immune system (80). The main targets of this system are non-species or non-mammalian molecules, among which advanced glycosphingolipids are important. In contrast, the T cells from the thymus-dependent immune system are also directed against intraspecies molecules that are foreign to the individual and which include cancer epitopes, the appearance of which is a large problem in species with long lifetimes. The side effect of this non-self guardian is that, occasionally, T cells show aggression toward self-tissue, and thereby potentially create an autoimmune disease. However, it seems that the more cells of the immune system other than T cells are demanded, the less likely is the risk of autoimmunity. It might be beneficial to have a certain amount of recurrent infections in which T cells are involved and occupied i.e. by antibody production. Thus, children attending pre-school day care, a proxy measure of total exposure to infectious diseases in early childhood, were found to have a lower incidence of T1D, with a pooled odds ratio of 0.59 (81, 82).

In autoimmune diseases the hygiene hypothesis has been established (83). First formulated by Strachan in 1989, it stated that autoimmunity is more common in clean surroundings and less frequent when the organism is well stimulated by microbes. The mechanism for this seems to be involvement of the innate immune system. The reaction against glycolipids, mainly but not exclusively those produced by microbes, is performed by this innate immune system. The glycolipids are taken up by dendritic cells and presented to NKT cells. These are divided into invariant NKT (iNKT) or type 1 NKT cells and non-iNKT or type 2 NKT cells. The former type are defined by their reaction to α-galactosyl ceramide (α-GalCer) whereas the non-iNKT cells react with other glycolipids including the mammalian β forms such as sulfated galactosyl ceramide (sulfatide) (84).

Glycosphingolipid molecules cannot be presented by the MHC complex, which only binds peptides, but presentation is achieved through the MHC-like CD1 molecules (85). The CD1 molecule contains a groove with two large hydrophobic pockets that are able to anchor the lipid tails of a glycosphingolipid (86). The human dendritic cells can express five kinds of CD1 molecules. These are divided into two groups: group 1 includes CD1a, b, c, and e, and group 2 is comprised of CD1d, which is the only one that mouse cells express.

Treatment of mice with sulfatide prevents antigen-induced experimental autoimmune encephalomyelitis, which is an animal model of human multiple sclerosis (26). Sulfatide had no effect in CD1d-deficient mice, indicating that the protective effect of sulfatide involved binding to CD1 (26). Actually, the structural basis of the CD1 presentation of sulfatide is well known, with two pockets for the fat tails of the glycolipid (87, 88). The most investigated CD1 ligand is α-GalCer, which is isolated from marine sponges. Both SJL and NOD mice, animal models of autoimmune diseases, have defects in NKT cell development and/or function (89, 90), and in humans with autoimmune diseases NKT cell numbers are reduced (91). α-GalCer-specific activation of iNKT cells protects against diabetes in NOD mice (92–94), and such protection has also been shown by CD1d restricted type 2 NKT cells in transgenic mice (95). This might as well be the mechanism for the sulfatide prevention of T1D in NOD mice (27). Overexpression of NKT cells protects transgenic NOD mice from diabetes (96), whereas a shortage of NKT cells in CD1d knock-out mice leads to exacerbation of type 1 diabetes (97). Finally, upregulation of CD1d expression within the beta cells restores the immunoregulatory function of NKT cells and prevents diabetes in NOD mice (98). It has been demonstrated that NKT cells inhibit the onset of diabetes by impairing the development of pathogenic T cells specific for pancreatic beta cells (99). This inhibition of T cell differentiation into effectors by NKT cells seems to require cell contacts (100). Even for inhibiting secondary enchephalomyocarditis (EMC) virus infection CD1d molecules are important (101). The mechanisms for this include activation of NKT cells and better production of interferon-alpha (101).

### Suggestions for treatment

The implications according to the hygiene theory are to avoid too clean surroundings in order to minimize the incidence of T1D (102). Also, not to treat aggressively cases of parasites (innocent pinworms etc) in children since these may lower the risk of developing T1D (103). Treatment with probiotics has been suggested, but no final human results exist so far (104). Allergen induction of a minor eczema, which seems to facilitate proliferation of NKT cells, reduces diabetes incidence in NOD mice, which is likely to reflect the decreased risk of T1D in humans with allergic dermatitis (105). Treatment with α-GalCer might be considered, but caution regarding the effect on other organ systems may be the reason for the lack of human trials. Sulfatide is a compound to consider for future investigations (27).

The *risk* of treatment influencing NKT cells is to disturb the delicate balance between type 1 and type 2 NKT cells (84) although this area for tumor immunology seems not to be finally established (106).

## IV. The importance of the intestine and gliadin intake

In 1993 we discovered that hydrolyzed diet protects against T1D in NOD mice (107). It was new and not generally accepted that changes in the diet could influence the incidence of diabetes. Six years later, the study was extended more specifically to gluten-free diet, which lowered the T1D incidence from 64% in the chow-fed control NOD mice to 15% in the experimental mice (108). In a more recent study in which the gluten-free diet-treated animals had never experienced gliadin, not even in fetal or neonatal life, the decline in incidence of T1D was from 61% to just 6% (109). Such a dramatic decrease is hardly seen otherwise and would demand more or less toxic procedures (e.g. ablation of T cells) that are not realistic for human use. Later, the same preventive results using gluten-free diet were obtained in BB rats (110) and by other groups also in NOD mice (111, 112). In humans, a time window was described for the optimal introduction of wheat in postnatal life (113, 114); this should be between the age of four and six months, otherwise the risk of beta-cell autoimmunity increased up to 4 times. Interestingly, in principle the same has been found for BB rats (115).

Gluten is composed of glutenin and gliadin. It is strongly hydrophobic, which is a desirable property for keeping white bread together. Mankind has known gluten for only 10,000 years, when we began our agricultural way of life in Mesopotamia. Since then wheat has been further refined and has been used more and more. Different sorts of wheat exist, and breads are different in structure when comparing Southern Europe to Scandinavia, where the composition is relatively compact. The degree of hydrophobicity may also vary, but in any case it is difficult to dissolve gluten in the intestine, which is necessary for the enzymes to operate and to break down the molecules. The result is that parts of undigested gliadin molecules irritate the intestinal mucosa, inducing unspecific, subclinical inflammation. Compared to conventional food, a gluten-free diet increases the amount of regulator T cells in Peyer's patches in the intestine (116).

Some people have special problems with gliadin and develop gluten intolerance in the form of celiac disease. Up to 10% of patients with T1D also have celiac disorder and, interestingly, the two diseases are by far most commonly seen together if diabetes is the first one to appear, and seldom if celiac disease develops first and a protective gluten-free diet is implemented (117). The symptoms of celiac disease are abdominal pain and diarrhea, which disappear when the patient stops the intake of gluten. If not, the patient will display enteropathy with atrophic villi and infiltration of immune cells in the intestine. The celiac patients display antibodies against tissue transglutaminase (tTG) even before clinical symptoms develop. They often share risk HLA tissue types (HLA-DR3) with T1D patients, but this is not the entire explanation for the co-morbidity.

NOD mice fed on a gluten diet also display intestinal enteropathy (112) and also have tissue transglutaminase (tTG) antibodies (118). BB rats have impaired intestinal function (119) and increased intestinal permeability (120). This is known in pre-T1D humans as well (121). Gliadin increases zonulin expression, and thereby gut permeability (122). Also enterovirus increase the intestinal permeability (123). The consequence of this may be higher uptake of bacteria toxins like LPS, and that molecules of partly digested gliadin pass the intestinal barrier. Indeed, non-degraded gliadin has been demonstrated in breast milk from healthy mothers (124). The transport must be mediated through the blood, which means that also other organs with a relatively heavy blood flow such as the islets of Langerhans could in principle experience gliadin and perhaps even gliadin deposits.

If there are gliadin deposits in the islets, brought by the bloodstream, this might be of special interest since diabetogenic T cells are primed both in pancreatic and gut-associated lymph nodes in NOD mice (125). In this connection, it is worth noting that in BB rats, before insulitis is established, the mesenteric lymph nodes of wheat-fed rats contain an unusually high proportion of Th1 cells that proliferate specifically in response to wheat protein antigens (126). If the gliadin is present in the islets, these T cells directed against wheat might give rise to the first tiny insulitis process. As intranasal administration of gliadin downregulates the immune response to wheat gliadin, as shown in DQ8 transgenic mice (127), we suggest treatment using nasal gliadin to stop insulitis and thereby diabetes.

It might be presumed that when gluten-free diet protects against diabetes, excess of gluten intake would accelerate development of diabetes. This is not the case; in contrast, it inhibits T1D as well (109, 128). The reason for this paradox is unknown, but the effect might be speculated to be due to an LPS-like stimulation of Toll-like receptor (TLR) 4, which is known to inhibit diabetes.

Regarding human studies, 6 months of gluten deprivation do not influence humoral autoimmunity, but may have a beneficial effect on preservation of beta-cell function in subjects at risk for T1D (129). Also, a study lasting 12 months in young non-diabetic children who were first-degree relatives of T1D patients showed no effect on diabetes incidence 5 years later (130). This is in good agreement with our experience using NOD mice, that shorter term gluten-free treatment has no effect on later diabetes incidence when the animals are again fed a gliadin-containing diet (Funda DP et al., unpublished). A time period with gluten-free intake does not pay off later in life; the diet works only when it is instituted.

Gluten is known to influence the composition of the bacterial gut flora (131). The flora can also be manipulated by probiotic administration, which can prevent spontaneous autoimmune diabetes in NOD mice (132). Furthermore, antibiotic treatment changes the intestinal distribution of bacteria, and in the first study on this regarding T1D, using fusidic acid we could indeed reduce the incidence of diabetes in BB rats (133, 134). This has later been confirmed with other antibiotics as well (135). Treatment with antibiotics may diminish the amount of bacteria and thereby the concentration of endotoxins. This may increase insulin sensitivity. We actually found lower blood glucose levels in non-diabetic rats that were given fusidic acid (136). The presence of certain bacteria is likely to be influenced by antibodies to special blood groups, as these Abs may be directed against mutual glycosylated epitopes; this might be the explanation for the reduced frequency of T1D in Lewis a-b- individuals (137). The bacterial colonization after birth is important for the expression of MHC class II molecules; the sooner this takes place, the better the definition of self. Delayed colonization has been suggested to be the reason for the 23% increased risk of T1D after birth by cesarean section compared to vaginal delivery (138).

### Suggestions for treatment

The effects of a gluten-free diet include less inflammation in the intestine, more regulator T cells in Peyer's patches, changed bacterial composition, a less permeable intestinal barrier, and possibly less gliadin molecules in the blood; all in favour of inhibiting T1D development. A gluten-free diet is not easy to implement, however, and as mentioned, short-term gluten-free intake has no effect. For this reason, more specific treatments have been suggested. These include injections with zonulin receptor mAb (139, 140), which should reduce gut permeability. Also, treatment with probiotics or antibiotics might be considered, but none of these suggestions seem close to being tested in human trials.

## Other factors related to development of T1D that are difficult to manipulate

### Genetics

The genetics of T1D is not the scope of this review, but it should be stressed that to a certain degree, the risk of developing T1D is influenced by genetic factors. In a Finnish study, the probandwise concordance was 42.9% for monozygotic and 7.4% for dizygotic twins (2). About twenty chromosomal regions are known to carry risk genes, and among these is by far the strongest the HLA region with a predicted odds ratio of 6.8 (141). Interestingly, these genes mirror the reaction to foreign antigens. The gene for insulin (INS) displays the second highest odds ratio of 2.3, then comes the immune-related lymphoid protein tyrosine phosphatase (LYP) with 2.0 and IL2 receptor alpha (IL2RA) with 1.5, whereas all other genetic risk regions have odds ratios of less than 1.25 (141). As the incidence of T1D has increased over the last decades, the frequencies of high-risk HLA types have declined, or in other words, the high-risk genes have been diluted among T1D patients (142).

As it is now, the DNA code for each individual is given and cannot be manipulated. The specific sequence is interesting to know only for evaluation of the risk of developing the disease. However, for the individual person the risk is seldom high enough to justify specific precautions, as described earlier in this review. For this purpose presence of autoantibodies is much stronger. This might be changed due to the growing area of epigenetics.

### Circumstances in fetal life

Many diseases are influenced by the fetal and perinatal life. In T1D, two studies have found a slightly shorter length of pregnancy in mothers of boys who later develop the disease (143, 144). Also, higher age of the mother (143) and higher birth weight predispose for T1D with an increased risk of 6–10% (145). For individuals who are born by cesarean section, the risk of acquiring T1D later in life is increased by 23% (146), which is actually more than the predisposition of most genes. It is unknown whether the mentioned risk factors are associated with differences in beta-cell volume. This varies with a magnitude of two to three among commonly used mouse strains, and may relate to different diabetes susceptibilities (49, 147).

### Virus in beta cells

Several studies indicate that enterovirus is frequently present in newly diagnosed T1D patients (14, 148–151). Dotta et al. has found that 3 of 6 T1D patients, but none of 26 controls, had evidence of Coxsackie B4 virus in their beta cells (14). If these kinds of slow virus are not destroyed by the innate immune system, including NK cells, interferon α (IFNα), or induction of apoptosis of the cells involved, then through class I presentation the virus will continue to stimulate the acquired immune system, which will finally attack the beta cells involved. Even though beta cells compared to alpha cells may have an especially strong response of 2′,5′A synthetase, the products of which, 2′,5′-oligoadenine nucleotides, activate mRNA degrading enzymes (152), the virus might not be eliminated and the mentioned events may take place.

If it turns out that only a few virus strains are responsible for infection of beta cells, vaccinations against these might be a possibility. Otherwise, strategies in order to avoid enterovirus infections seem to be unrealistic.

## Etiologies and pattern of progression to Type 1 diabetes

A good deal is known about the pathogenesis of T1D, but no firm knowledge exists about the etiology of the disease. As disorders are initiated by something, such “something” will now be suggested and events will be speculated.

Traditionally, *enterovirus* has been suggested as the etiological factor. Indeed, virus exist that can induce diabetes in mice dependent on the immune system (8) as demanded by the current pathogenetic understanding as mentioned above. Several different virus can induce T1D in animal models, but in humans especially the Coxsackie virus have been considered. T1D does develop more frequently in the autumn when enterovirus are common, and the recent findings of somewhat silent enterovirus in the islets of newly diagnosed T1D patients (14) are highly interesting etiologically.

Also, beta-cell toxins have been considered etiologically. In mice, streptozotocin can induce T1D, even in low multiple doses (153) which works via an immunological mechanism (154). However, for the vast majority of T1D patients no direct beta-cell toxic compound has been identified. An apparently non-toxic compound has to be considered instead.

*Gliadin* has, as mentioned in point IV, an important impact on T1D development, and to the best of our knowledge this is not the case for any other external compound. Therefore, gliadin is hereby suggested as an etiological agent for T1D, not in the sense like an infectious agent to be the direct cause of a certain disease, but rather as a starter of a long, complicated process leading to T1D.

### The following scenario can be hypothesized

Virus inside a beta cell cannot be destroyed by immune cells, but it can disappear due to interferon and 2′,5′A synthetase dependent RNAse activity or due to apoptosis of the host cell. Being in the blood, virus can be neutralized by antibodies. Thus, for elimination of a virus, apoptosis is a beneficial process. Of course, beta cells are lost, but the huge (re)generation capacity can just create some new ones. This is seen during pregnancy (155), and as long as the beta cells are not attacked by the immune system or are toxically influenced by high glucose or high fat concentration or insulin resistance, new beta cells are generated. In support of the benefit from apoptosis in T1D development is the fact that the best remission period is seen in T1D patients with a high level of IL-6 (156) and/or a low level of adiponectin (157). In contrast, both low concentrations of IL-6 and high amounts of adiponectin inhibit apoptosis and are desirable in T2D. Furthermore, the vitamin D level does not influence the development of T1D (Norris JM, personal communication) whereas high concentrations protect against T2D. Vitamin D and its analogs have been shown to inhibit apoptosis in beta cells after cytokine exposure (30). In as much as apoptosis might be valuable in T1D, this is most likely not the case in a degenerative disease like T2D (158). Furthermore, it should be stressed that these considerations cannot be paralled to the commonly used T1D animal models, which do not have a viral etiology. Patients developing T1D have increased intestinal permeability (121), which likely leads to a higher level of LPS in the blood; due to a TLR4 mechanism, this will inhibit apoptosis (159) being unbeneficial for a virus-containing beta cell.

Simultaneous with the intracellular virus deposits, gliadin is eaten but not fully digested and some molecular parts are penetrating to the bloodstream. Among other organs (including the lactating breast (124)), gliadin is likely brought to the highly vascularized islets and might to a minor degree adhere to them. The uptake from the intestine might be caused by a zonulin-induced increased permeability in diabetes susceptible individuals (140) and/or might be due to infection with any enterovirus, which causes a more leaky intestinal barrier (123). In the islets, dendritic cells are activated by the gliadin molecules, which are presented to T cells in the regional lymph nodes. These also drain the intestine (125), and experienced T cells directed against gliadin might fuel the process and start an insulitis reaction. From the islets’ point of view this insulitis is unspecific, but it might also draw attention to the possible present enterovirus, and a more serious insulitis inflammation in the islets will take place. If this is repeated and is lasting for some time, activated monocytes (160) may be tasting not only gliadin but also necrotic beta cells infected with virus. Then a class II process against the virus will be displayed. Due to immunological exposure after perforin/granzyme attacks, this might give rise to immune reactions against specific, important beta-cell antigens such as insulin, GAD, zinc transporter etc. So if not by apoptosis, human beta cells are destroyed by a necrotic process, which facilitates antigen spreading. This might be helped along by high beta-cell activity (fever, intake of refined carbohydrates), by a reactive thymus-dependent immune system with suppressed regulator function, and by relatively few NKT cells.

The class II immunity might be the key event. Administration of silica, which inactivates macrophages, prevents T1D in BB rats (161). Actually, in NOD mice CD4 cells are necessary for T1D development even more than CD8 cells (162, 163). Injection of molecules from the relevant tissue together with Freund's adjuvant in order to raise a class II immune reaction can induce autoimmune diseases. By such a procedure, e.g. experimental allergic encephalitis (EAE) can be introduced in animals, but T1D cannot. This might be due to strong regulator T cell reactivity against insulin and other beta-cell antigens. But as hypothesized, gliadin might provoke a class II immune reaction *in situ* of the islets, and this might also be the case for hidden virus after necrosis of beta cells, as described.

For the virus part, an analog to herpes zoster may be speculated. Here, the etiology is a slow virus, concealed in a sensory ganglion. This virus can be activated, often in association with a suppression of the immune system e.g. due to pregnancy or leukemia. A lower antibody titer might facilitate presentation of the virus particles to the CD4 effector T cells and a cellular inflammation will take place. Interestingly, T1D patients have lower antibody titers against Coxsackie virus than healthy controls (164, 165). During this possible class II immunity against virus, interferon α will be activated. This is actually found to be expressed in pancreases of T1D patients (166), and it is known to induce T1D in transgenic mice (167). Through IFNα’s activation of the especially strong response of the 2′,5′A synthetase system in the beta cells, these and not the alpha cells are hurt by the mRNA degrading enzymes, which may explain the beta-cell specificity of T1D (152). Furthermore, T cells in the insulitis process might call for NK cells. When present *in situ,* these cells attack the beta cells due to their ligand to NKp46, which is not present on alpha and delta islets cells (168). In a cell-to-cell contact, the NK cells degranulate into the beta cells (168).

At this stage, various amounts of beta-cell antibodies are present (169), but the process may still be reversible. However, after repeated similar attacks more and more effector T cells are raised and more and more beta cells are destroyed (170), and a point of no return is passed. The insulitis process perpetuates by itself and clinical diabetes will occur. At the time of diagnosis of T1D, no treatment is known to be able to cure the disease since the T cell immune reaction is heavy and irreversible.

## Final remarks

T1D is a complicated disease that is difficult to understand; the question of what causes T1D is still not fully answered, but much is known. Our present knowledge would not have been obtained without the use of animal models. The lessons are that T1D will not develop unless the four numbered pathogenetic factors (related to: T cells, beta-cell activity, NKT cells, and the intestine) act in concert to some degree, and that if any of the four factors are neutralized, inhibited, or acted against, T1D will not occur.

## Conflicts of interest

The author declares no conflicts of interest.

## Dansk resumé

Afhandlingen omhandler den viden dyremodeller har skabt omkring årsagerne til Type 1 diabetes (T1D). Uden eksistensen og brug af dyremodeller ville den nuværende forståelse af sygdommen være på et væsentligt lavere niveau, og behandlingsforsøg og –tilbud være betydelig mindre udviklede. Forekomst af T1D er til dels bestemt af genetiske faktorer, men med en tvillingekonkordans på 30–40% er miljøfaktorer af stor betydning.

Vedrørende patogenesen til T1D er fire områder specielt vigtige: 1) Det thymus-afhængige immunapparat: T1D er en T celle drevet sygdom og beta-cellerne ødelægges gennem en lokal betændelse, kaldet insulitis. Der er tale om en autoimmun proces med brud på selvtolerance, hvor regulator T celler søger at standse de aggressive effektor T celler. Hæmning af T celler (fx med anti-CD3 antistoffer eller med cyclosporin) vil bremse T1D processen selv hvis den er initieret af virus. Den teoretiske risiko ved immunmodulerende behandling er en højere frekvens af malignitet. 2) Aktiviteten af beta-celler: Hvilende beta-celler udviser i mindre grad antigenicitet end aktive og er mindre følsomme for immundestruktion. Beta-celle hvile kan opnås ved at indgive insulin, så behovet for egenproduktion mindskes, eller ved behandling med kalium-kanal aktivatorer. Begge procedurer forebygger T1D i dyremodeller, mens der ikke findes gode humane undersøgelser til dels på grund af frygt for hypoglykæmi. 3) NKT celler: I henhold til hygiejne teorien vil stimulation af NKT celler med ikke-patogene mikrober give anledning til mindre T celle reaktion og dermed mindre autoimmunitet. Væsentlig for denne stimulation er glycolipider præsenteret af CD1 molekyler. 4) Betydningen af gluten indtag og tarmforhold iøvrigt: Gluten-fri diæt forebygger dramatisk T1D i dyremodeller, og epidemiologiske data støtter en effekt hos mennesker. Mekanismerne inkluderer mindre subklinisk betændelse og permeabilitet i tarmen, samt en ændret tarmflora der også kan opnås ved indtag af probiotika. Gluten-fri diæt er vanskelig at implementere og korttidsbehandling har ingen effekt.

Vedrørende selve starten af T1D processen spekuleres enterovirus og gliadinaflejringer at have ætiologisk betydning i genetisk følsomme individer, hvorefter de fire nævnte patogenetiske faktorer i en grad af forening er aktive i T1D sygdomsudviklingen. Læren fra dyremodelstudier er at neutralisation af en hvilken som helst af de fire patogenetiske faktorer kan stoppe T1D sygdomsprocessen.
